# S/N/O-Enriched Carbons from Polyacrylonitrile-Based Block Copolymers for Selective Separation of Gas Streams

**DOI:** 10.3390/polym16020269

**Published:** 2024-01-18

**Authors:** Diego Gómez-Díaz, Lidia Domínguez-Ramos, Giulio Malucelli, María Sonia Freire, Julia González-Álvarez, Massimo Lazzari

**Affiliations:** 1 Departamento de Ingeniería Química, ETSE, Universidade de Santiago de Compostela, Rua Lope Gómez de Marzoa s/n, 15782 Santiago de Compostela, Spain; lidia.dominguez2@usc.es (L.D.-R.); mariasonia.freire@usc.es (M.S.F.); julia.gonzalez@usc.es (J.G.-Á.); 2 Departamento de Química Física, Facultade de Química, Universidade de Santiago de Compostela, Avenida das Ciencias s/n, 15782 Santiago de Compostela, Spain; 3 Centro Singular de Investigación en Química Biolóxica e Materiais Moleculares (CiQUS), Universidade de Santiago de Compostela, 15782 Santiago de Compostela, Spain; 4 Department of Applied Science and Technology, Politecnico di Torino, Viale Teresa Michel 5, 15121 Alessandria, Italy; giulio.malucelli@polito.it

**Keywords:** block copolymer templates, hierarchical pores, atom transfer radical polymerization, sulfur-doping, oxidative-stabilization

## Abstract

A series of polyacrylonitrile (PAN)-based block copolymers with poly(methyl methacrylate) (PMMA) as sacrificial bock were synthesized by atom transfer radical polymerization and used as precursors for the synthesis of porous carbons. The carbons enriched with O- and S-containing groups, introduced by controlled oxidation and sulfuration, respectively, were characterized by Raman spectroscopy, scanning electron microscopy, and X-ray photoelectron spectrometry, and their surface textural properties were measured by a volumetric analyzer. We observed that the presence of sulfur tends to modify the structure of the carbons, from microporous to mesoporous, while the use of copolymers with a range of molar composition PAN/PMMA between 10/90 and 47/53 allows the obtainment of carbons with different degrees of porosity. The amount of sacrificial block only affects the morphology of carbons stabilized in oxygen, inducing their nanostructuration, but has no effect on their chemical composition. We also demonstrated their suitability for separating a typical N_2_/CO_2_ post-combustion stream.

## 1. Introduction

Block copolymers (BCs) have emerged since the 90s as a pivotal class of polymeric materials suitable for a wide range of applications, from additives for polymer blends to drug delivery and nanotechnology, including energy storage and conversion systems [1,2,3,4]. These copolymers consist of two or more chemically distinct homopolymer fragments, usually immiscible into each other, joined together by covalent bonds, thus resulting in a series of fascinating periodical nanophase-separated morphologies [5]. Strict control of the molecular weight distribution of each polymeric component and their self-assembly allows obtaining periodical nanofeatured structures that are widely proposed as nanomaterials themselves or as a template for the fabrication of nanomaterials or part of devices after different processing, such as thermal treatments, selective degradation of some components or nanolithography, among others [6,7].

Polyacrylonitrile (PAN) is the most commonly used industrial precursor for the fabrication of carbon fibers due to its superior strength and stability and higher carbon yield. Using copolymers based on PAN takes advantage of these peculiarities, combining the inherent carbonization ability of PAN with the versatility of BCs, thus aiming for precise structural control over the final carbon product [4]. This design flexibility is expected to enable fine-tuning of the resulting carbon material, i.e., influencing critical factors like pore size distribution, surface area, and electrical conductivity.

Matyjaszewski’s group first prepared N-enriched carbons from well-defined PAN-based block copolymers with poly(*n*-butyl acrylate) (PBA) as a sacrificial block [8,9]. The resulting hierarchical porous structures, as well as the carbons obtained from polyacrylonitrile-*block*-poly(methyl methacrylate)s PAN-*b*-PMMA [10], only showed limited preservation of the initial BC phase-separated nanostructures. They were proposed to fabricate supercapacitors with unusually high capacitance per unit surface area, possibly attributed to the pseudocapacitance due to the high nitrogen content originating from the PAN precursor [8]. More recently, Yuan et al. demonstrated that sulfur stabilization during the synthesis of PAN-*b*-PBA-templated N-enriched carbons improved their alkaline supercapacitor performance. In addition, the mesopore size optimization was enabled by controlling the block length of the copolymer precursor [11].

Further studies focused on the synthesis of porous carbon fibers from different PAN-based BCs, exploring a wide range of BC molecular weight and composition, i.e., with PAN block number-average molecular weights (*M_n_*) between 13 and 215 kDa and PAN volume fraction (φ_PAN_) between 0.25 and 0.75, and PAN *M_n_* of 101 kDa and φ_PAN_ 0.84 in PAN-*b*-PMMA [12] and polyacrylonitrile-*block*-polystyrene (PAN-*b*-PS) [13], respectively. Interestingly, BCs formed disordered and kinetically trapped nanostructures after electrospinning and thermal treatment instead of the expected classical BC morphology, such as spherical, cylindrical, and lamellar structures. On the other hand, regardless of the φ_PAN_, all the precursors were pyrolyzed into disordered and interconnected porous carbons, with mesopores that became smaller while increasing φ_PAN_ content. BC, with a PAN volume fraction near 50%, achieved the highest surface area and the largest gravimetric capacitances [12].

On such a basis, considering the use of carbons fabricated from PAN homopolymer for CO_2_ separation by gas-solid adsorption [14], we investigated the use of PAN-*b*-PMMA BCs as precursors in this work. To this aim, a series of BCs with a common PAN block with low molecular weight and different BC compositions were proposed to synthesize carbons with increased porosity in the mesoporosity range. Doping of carbons with N, S, and O, which induce atomic charge density and/or spin density redistribution, is expected not only to increase their specific capacitance [8,11,15] but was also reported to favorably enhance electrocatalytic activity [16] and more closely to the specific aim of this work, adsorption selectivity of heavy metals [17] and, e.g., gas separation [18]. With such rationale in mind, we explored the effect of N-containing groups and O- and S-containing groups, introduced by controlled oxidation and sulfuration, respectively, on the adsorption capacity of our carbons. In addition, we studied the relationship between the BC composition, i.e., φ_PAN_, and the carbon morphology and adsorption characteristics, trying to disclose further details on the mechanism of carbon formation.

## 2. Materials and Methods

### 2.1. Polymer and Carbon Synthesis

Acrylonitrile (Merck, Darmstadt, Germany, 99%) was passed through a macroreticular ion exchange resin (De-Hibit-200, Polysciences Inc., Warrington, PA, USA) to remove the inhibitor. Methyl methacrylate (99%), 2-bromopropionitrile (BPN, 97%), CuBr (98%), 2,2′-bipyridine (99%), N,N,N′,N′,N″-pentamethyldiethylenetriamine (99%), N,N-dimethylformamide (DMF, 99.8%), 2-cyanopyridine (2CNP, 99%), ethylene carbonate (EC, >99%), sulfur (>99%, rhombic sulfur), methanol, and 2-propanol were purchased from Merck and used as received. 

All the polymer syntheses were carried out by copper-mediated atom transfer radical polymerization (ATRP) in EC or 2CNP, using BPN or bromine-terminated PAN as initiators for homopolymerization and copolymerization, respectively, following previously reported procedures [19,20]. At the end of the polymerization, the reaction mixture was dissolved in DMF, and the polymer was precipitated in a methanol/2-propanol (3/1 *v*/*v*) mixture. The polymer was filtered, washed several times with methanol and water, dried under vacuum to constant weight, and finally purified, passing its DMF solution through alumina columns to remove Cu and other residues. Carbon was synthesized in a tubular furnace (Carbolite, Sheffield, UK) operating under O_2_ or N_2_ flow.

### 2.2. Polymer and Carbon Characterization

Composition and end-group analyses of polymers were carried out using ^1^H NMR in DMF-*d*_7_ at 353 K on a DRX-500 MHz spectrometer (Brucker, Billerica, MA, USA). Thermogravimetry (TGA) measurements were performed with a Q5000 IR thermobalance (TA Instruments, New Castle, DE, USA), operating under 50 mL min^−1^ N_2_ flow. 

Raman spectra were obtained using an alpha300 R+ Raman Microscope (WITec, Ulm, Germany) with a 532 nm laser and a 600 g/mm grating. A Zeiss EC Epiplan-NEOFLUAR 50×/0.8na objective was used, with integration times spanning from 0.1 to 0.5 s and the number of accumulations from 100 to 500. Laser power was between 0.5 and 2.5 mW. Imaging processing, including spectral deconvolution, was performed with WITec Suite FIVE version 5.1 software.

The chemical nature and atomic quantity of the functional groups of carbons were studied by X-ray photoelectron spectrometry (XPS). A PHI 5000 Versa Probe instrument (Physical Electronics, Chanhassen, MN, USA) was equipped with an Al Kα radiation (1486.6 eV) X-ray to analyze the XPS. The morphological and structural features were assessed by field emission scanning electron microscopy (SEM) using a Fesem Ultra Plus (Zeiss, Jena, Germany), working at 3 kV.

An ASAP 2020 adsorption volumetric analyzer (Micromeritics Instruments, Norcross, GA, USA) was used to determine the surface textural properties of carbons prepared in the present work, such as surface area, pore volume, and average pore size. These data were determined based on experimental data corresponding to nitrogen (77 K) and carbon dioxide (273 K) adsorption. In a previous step, carbons were outgassed under vacuum at 573 K for 2 h with a heating ramp of 10 K min^−1^. For both gases, the BET model was employed to estimate the specific surface area, and total pore volume was determined at a relative pressure of 0.99.

The same experimental setup was employed to determine nitrogen and carbon dioxide adsorption isotherms at different temperatures (273–323 K) and pressures (0–760 mmHg). The same outgassed procedure that was previously described was utilized before adsorption experiments. An ice bath in a Dewar was employed to determine gas adsorption isotherms at 273 K, while a temperature control water bath using a Selecta Sensoterm controller was used for experiments at 298 and 323 K.

Gas adsorption experiments for surface characterization and gas separation studies were carried out in duplicate to confirm the suitability of the collected experimental data.

## 3. Results

### 3.1. Synthesis and Structural Characterization

A series of PAN-*b*-PMMA diblock copolymers were synthesized by ATRP using a common PAN macroinitiator, following a procedure that assures good control over the relative block lengths [19,21]. BC compositions were selected to obtain different phase-separated morphologies, i.e., spherical, cylindrical, and lamellar structures with PAN as the minority block [22] for BC10/90, BC26/74, and BC43/57, respectively. Polymeric precursor characteristics are collected in Table 1. Due to the high value of the Flory-Huggins interaction parameters in the PAN-PMMA system, high molecular weight values are unnecessary to take advantage of BC peculiarities. A molecular weight of the PAN block of just 1.3 kDa is enough to induce phase separation [9,20], thus allowing morphologies with nanodomains as small as possible to be transformed in nanostructured carbons.

Carbons with N- and O-containing groups were synthesized in a tubular oven by treating BC powders under O_2_ flow to a slow heating, 1 K min^−1^, up to 553 K, followed by a 1 h stabilization and further heating up to 1073 K, 5 K min^−1^, under N_2_ flow, followed by 0.5 h stabilization and natural cooling down to room temperature. It is well-known that the first step stabilizes the structures formed by PAN cyclization, also introducing O-containing groups (the simplified mechanism is recalled in Scheme S1, in the supporting information [23]). The complete PMMA decomposition, essentially by unzipping [24], occurs during the second heating at higher temperatures, which also induces microporosity in the expected graphitic carbon [10]. S-containing carbons were synthesized from a 1/1 weight ratio of BC to sulfur mixtures through the same thermal treatment used for sulfur-free carbons but carried out under N_2_ flow to avoid easy sulfur oxidation and burning [10]. An illustrative chemical structure of the S-doped carbons is shown in Scheme S2 [25].

To quantify the pyrolysis behavior, the BCs and the corresponding homopolymeric components were analyzed by TGA under conditions that resemble those used for the large-scale pyrolysis realized in the tubular oven. Char yields are reported in Table 2 (the two series are named by adding the letter O or S after the acronym of the BC precursor, indicating when obtained through a stabilization under oxygen or in the presence of sulfur, respectively). As expected [8,20], the PMMA content of the precursor does not affect the carbon yield due to its complete volatilization. The char is only proportional to the PAN content, with values ranging from ca. 6% for both BC10/90-O and BC10/90-S to 24% for BC43/57-O. The partially graphitic nature of the carbons was confirmed by Raman spectroscopy. As already reported for the carbons prepared from PAN homopolymer [26], the spectra show the typical peaks centered at 1350 cm^−1^ (the so-called D band) and 1593 cm^−1^ (G band) associated with the breathing of C hexagons at the edges or the defects of the carbon structure, and to the in-plane vibration of *sp*^2^ of all graphitic C=C carbon forms [10,27], respectively (see, as examples, the spectra of BC10/90-O and BC10/90-S in Figure S1). G-line position is indicative of a high graphitization degree, while the intensity ratio of the height of the D band to the G band is inversely proportional to the graphite crystallite sizes [28]. It was estimated in the range of 0.88–0.95 for all the carbons, in line with the values obtained for carbons from PAN precursors [8,13,26]. 

**Figure 1:**
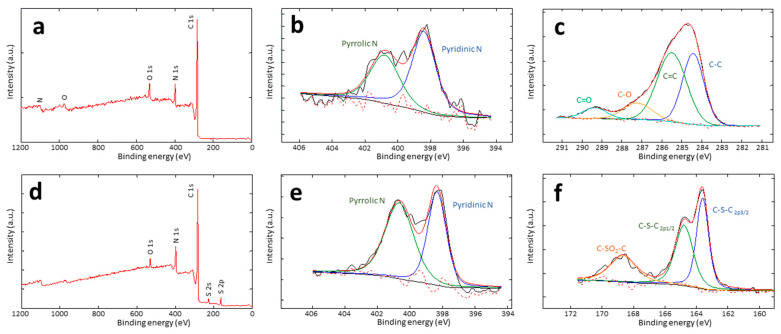
XPS spectra of BC26/74-O: survey (**a**), the high-resolution of N 1s (**b**), and C 1S (**c**); XPS spectra of BC26/74-S: survey (**d**), the high-resolution of N 1s (**e**), and S 2p (**f**).

The effective incorporation of heteroatoms within the carbon network, namely nitrogen from the PAN precursor, oxygen through oxidative stabilization, sulfur by direct addition, and the specific types of functional groups, were studied with XPS (Figure 1 and Table 3). N-containing structures consist of pyridinic (N 1s deconvoluted peak centered at 398.4 eV) and pyrrolic (at 400.8 eV) structures, eventually with small amounts of pyridine oxide groups (403.0 eV) [8]. The pyrrolic to pyridinic nitrogen ratio is 0.8–1.1 for both the BC-O and BC-S series. Concerning the bonds involving carbon in the BC-O series, the C 1s signal shows 4 deconvoluted peaks. Apart from the obvious peaks at approximately 284.4 and 285.5 eV corresponding to sp^2^ C bonding (C=C) and sp^3^C bonding (C-C), the two minor peaks at 287.2 and 289.4 eV can be attributed to carbons bonded to oxygen through a single or a double bond in ether-like (C-O) or carbonyls (C=O), respectively [29,30]. In addition, the signal of S 2p of BC-S carbons can be fitted into three peaks. The smallest, centered at 168.6 eV, corresponding to <10% of the total sulfur content in all the series, can be assigned to oxidized sulfur and is consistent with C-S(O)_2_-C sulphone bridges [31]. The two bigger peaks at 163.6 and 164.8 eV are due to the S 2p_1/2_ and S2p_3/2_ of C-S-C sulfide bridges, respectively [31]. 

We observed that the composition of carbons almost coincides with each other within the two series, with the content of C, N, and O of approximately 81, 13, and 6 wt.% for the BC-O carbons, and C, N, O, and S of 80, 12, 5 and 3 wt.% for the BC-S series, i.e., with a 19–21% total heteroatom content. As these values are also very similar to those already determined for carbons synthesized from PAN homopolymer as a precursor [26], likely, the presence of PMMA blocks with different lengths in the copolymers does not influence the chemical processes involved in the formation of the pyrolytic carbons. A similar behavior was observed for PAN diblock copolymers with poly(butyl acrylate) [11]. 

**Figure 2:**
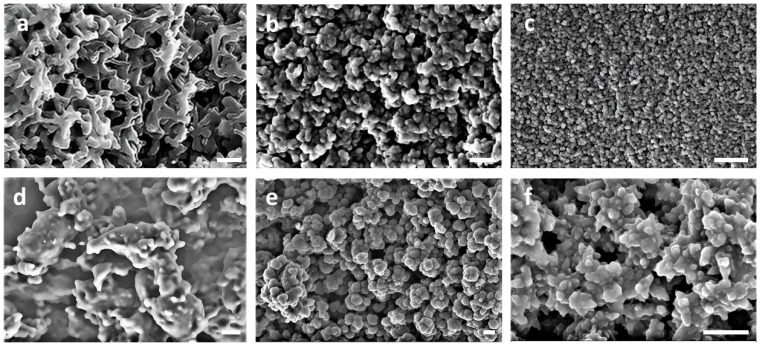
SEM images of BC10/90-O (**a**), BC26/74-O (**b**), BC43/57-O (**c**), PAN-O (**d**), PAN-S (**e**), and BC10/90-S (**f**). Scale bar 200 nm in all the images, except d (2 μm).

A direct visualization of the surface of carbons by SEM revealed their nanostructuration. The comparison of the BC-O carbons in Figure 2a–c with the more homogeneous appearance of carbon from PAN homopolymer (PAN-O) in Figure 2d confirms the role of PMMA as a sacrificial block to induce some porosity, at least in the mesoscale. In addition, an increase in the amount of PAN, i.e., in its volume fraction φ_PAN_, entails the formation of less defined pores. It is also clear that the BC-O carbon structures do not resemble the morphologies expected from conventional diblock copolymer self-assembling. Only in the case of BC10/90-O and BC26/74-O are carbon structures resembling that of a BC gyroid morphologies visible, although predictable only for BCs with a higher φ_PAN_. This apparent incongruity may be explained as follows, referencing the hypothesis proposed by Liu and co-workers [12]. Self-assembly of BCs into thermodynamically equilibrated morphologies may be reached through thermal annealing at a temperature above both the glass transition temperature, *T_g_*, and the possible melting temperature, *T_m_*, of all the BC components [32]. In the case of PAN-*b*-PMMA, the *T_g_* of both blocks is in the range 363–373 K, whereas the *T_m_* of PAN at 573–593 K [17] also corresponds to a temperature at which the polymer starts to decompose. Consequently, at the temperature of the stabilization step in oxygen, i.e., 553 K, the cyclization-oxidation of PAN fixes the BC morphology in metastable assembling before reaching the thermodynamically stable phase separation. The structure resulting from complete PMMA decomposition in the second step of carbonization depends on BC composition, and carbons with degenerated bicontinuous structures cannot be expected from compositions, potentially leading to spherical or lamellar morphology. Only BCs with φ_PAN_ in the approximate range of 0.10–0.30, potentially forming cylindrical or gyroid morphologies, seem good candidates for synthesizing carbons with interconnected pores [33,34]. 

The carbons synthesized in the presence of sulfur were very similar to each other and to S-doped carbon from PAN homopolymer, PAN-S (see some representative SEM images in Figure 2e,f). The condensed globular aggregates appear composed of large-sized spherical shapes with dimensions in the range of 50–100 nm. This evidence indicates the lower efficacy of sulfur with respect to oxygen to stabilize the developing morphologies. Metastable phases are not fixed as the presence of oxygen and carbon structures evolve further by a complete PMMA volatilization to a common pattern, regardless of the BC composition.

As a final consideration, SEM analysis also gives a first rough indication of part of the carbon mesoporosity (conventionally between 2 and 50 nm). Its quantification, as well as that of micropores (<2 nm), are usually carried out by adsorption/desorption experiments with nitrogen and CO_2_, respectively.

### 3.2. Surface Area and Pore Size

**Figure 3:**
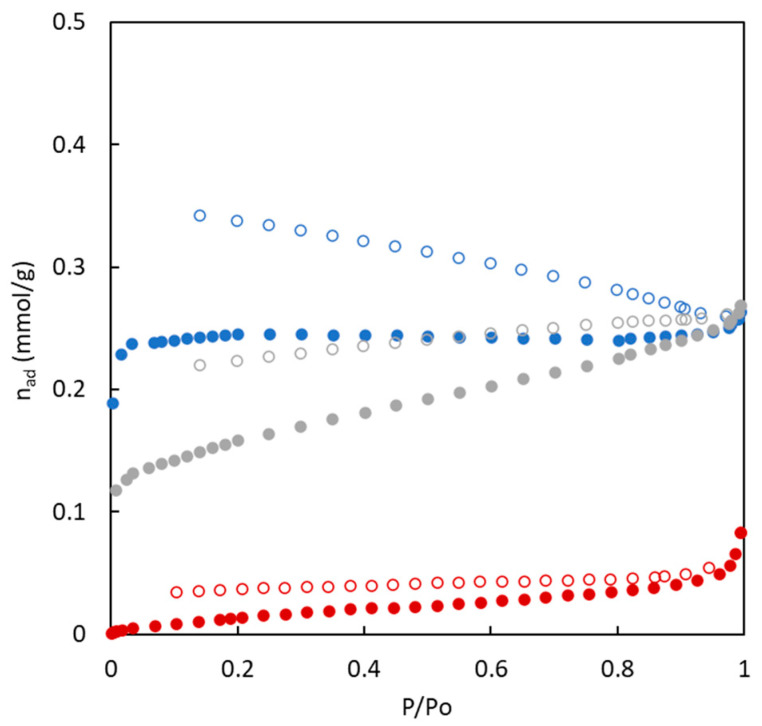
Adsorption (full symbols) and desorption (empty symbols) isotherms of N_2_ (77 K) for carbons from copolymers in the absence of sulfur. BC10/90-O (blue), BC26/74-O (grey), BC43/57-O (red).

**Figure 4:**
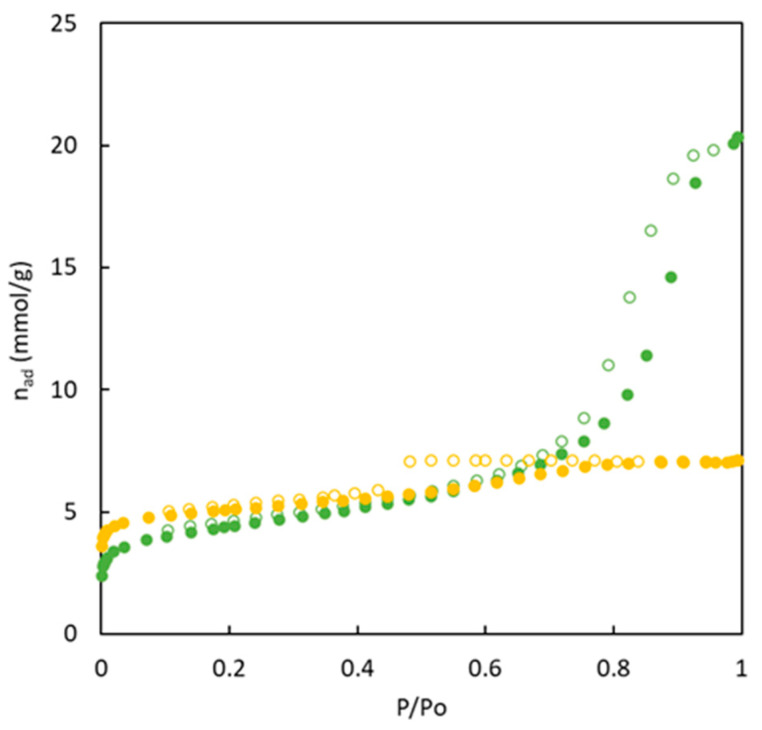
Adsorption (full symbols) and desorption (empty symbols) isotherms of N_2_ (77 K) for carbons from copolymers doped with sulfur. BC10/90-S (green), BC26/74-S (yellow).

Nitrogen adsorption/desorption isotherms at 77 K of the carbons synthesized without (BC-O series) and with (BC-S series) the use of sulfur are shown in Figure 3 and Figure 4, allowing us to obtain information about their porous structure. Certain differences are observed in the shape and magnitude of nitrogen adsorption isotherms. 

The experimental data corresponding to materials fabricated in the absence of sulfur showed that the amount of adsorbed nitrogen is low in all cases, but an increase in the amount of adsorbed nitrogen is observed with MMA composition. A slightly different shape of these isotherms is observed. Low MMA composition shows type IV isotherms (for BC43/57-O and BC26/74-O) related to a high presence of mesopores [35]. A higher presence of MMA (BC10/90-O) shows a type I isotherm characteristic of microporous materials. The isotherms classification for these materials agrees with the calculation of the microporosity percentage included in Table 2. An increase in MMA presence in the precursor tends to generate higher microporosity in the material. 

The analysis of the nitrogen adsorption/desorption isotherms for materials obtained in the presence of sulfur (see Figure 4) indicate important differences in comparison with the materials without sulfur. On the one hand, the amount of nitrogen adsorbed in the surface of the materials is higher for sulfur-doped materials in the entire relative pressure range. All these materials reach a first plateau close to 5 mmol g^−1^. At high relative pressure, adsorbed nitrogen is mainly enhanced for the BC10/90-S material. In addition, for these materials, a hysteresis loop is observed for all sulfur-doped materials. 

Considering the IUPAC classification of adsorption/desorption isotherms, the experimental data included in Figure 4 allows the inclusion of all isotherms in type IV. This type of shape of nitrogen adsorption isotherm at 77 K is characteristic of solids with an important mesoporosity. This type of solids and pore size (higher than micropores) tend to generate nitrogen multilayers when relative pressure increases (increasing the amount of adsorbed nitrogen). The first part of the isotherms at low relative pressure refers to the formation of the monolayer. In addition, the presence of a hysteresis loop is related to the nitrogen condensation in the mesopores. A decrease in the amount of MMA causes the opposite effect than for materials without sulfur, increasing microporosity and showing an important enhancement of nitrogen adsorbed by the formation of multilayers. The pore size distribution supports these conclusions about the type of pores in the different materials. For instance, Figure S2 shows the main presence of mesopores for BC10/90-S while BC10/90-O reaches very low values of pore volume. This type of analysis does not allow the analysis of the micropore range in detail. 

The experimental data corresponding to adsorption/desorption isotherms of N_2_ at 77 K can be used to analyze the porous structure of solids. Specifically, surface area and information about pore size have been included in Table 2. The calculated data show that the materials in the absence of sulfur reach very low surface area values (below 20 m^2^ g^−1^). On the other hand, using sulfur as a doping agent allows us to reach materials with a similar surface area (between 369 and 372 m^2^ g^−1^). The presence of sulfur atoms during carbon production accounts for an increase in surface area, which agrees with previous studies [29]. The influence of the BC composition in the precursor on the surface area value is evident only in the materials synthesized without sulfur. An increase in PMMA sacrificial block accounts for increased surface area caused by this polymer’s removal during carbon production. The results corresponding to carbons with sulfur do not show the influence of polymers’ composition. The overall behavior generally shows the following trend: BC10/90-S > BC26/74-S > BC10/90-O > BC26/74-O > BC43/57-O.

Nitrogen adsorption isotherms also allow us to calculate the porous volume of each pore type. Analyzing the materials in the absence of sulfur, very different types of pores are generated. For instance, a low PMMA content in the BC precursor appeared to generate only mesopores in the material, while a larger presence enhanced the generation of micropores. Though a lower presence of PMMA increases the pore size, from an overall point of view, an important decrease in the porous structure is observed.

The opposite behavior (in relation to the percentage of microporosity) was observed for sulfur-doped carbons. However, the presence of sulfur does not allow for reaching a high microporosity percentage because the presence of this type of atom tends to increase pore diameter [36]. In the same way as previously commented for materials synthesized in the absence of sulfur, a decrease in the amount of the sacrificial PMMA block composition tends to decrease the porous structure. However, the presence of sulfur increases the porous volume in all cases. 

All these results agree with the fact that the pore size in BC10/90-S is larger than in the other materials; further, it favors the formation of multilayers at high relative pressures, as observed in Figure 4. In the case of BC26/74-S, it shows a similar behavior at relative pressures lower than 0.7. However, the higher presence of microporosity avoids the sharp increase in nitrogen adsorption at high relative pressures. 

These experimental results and the differences observed depending on the copolymer composition allow us to conclude that the tuning of PMMA amounts promotes the synthesis of materials with a different degree of pore size and volumes. A comparison of present experimental data with those obtained for carbons from PAN homopolymers [22] indicates that the use of BC precursors only increases the surface area generated for supramicropores and mesopores (determined with N_2_ at 77 K) in sulfur-doped carbons and a slight decrease is observed for the other materials. 

Several studies [37,38] have concluded that the values for different parameters determined using the nitrogen adsorption experimental data at 77 K need to be revised to characterize carbons’ porous structure due to diffusion issues in micropores. For this reason, additional experimental data were obtained by replacing the adsorbate; in particular, carbon dioxide adsorption isotherms at 273 K were recorded for all the investigated materials. These previous studies concluded that this type of experimental data allows us to analyze microporosity deeply, specifically ultramicroporosity (pore diameter below 0.7 nm), where nitrogen molecules present diffusion limitations. 

**Figure 5:**
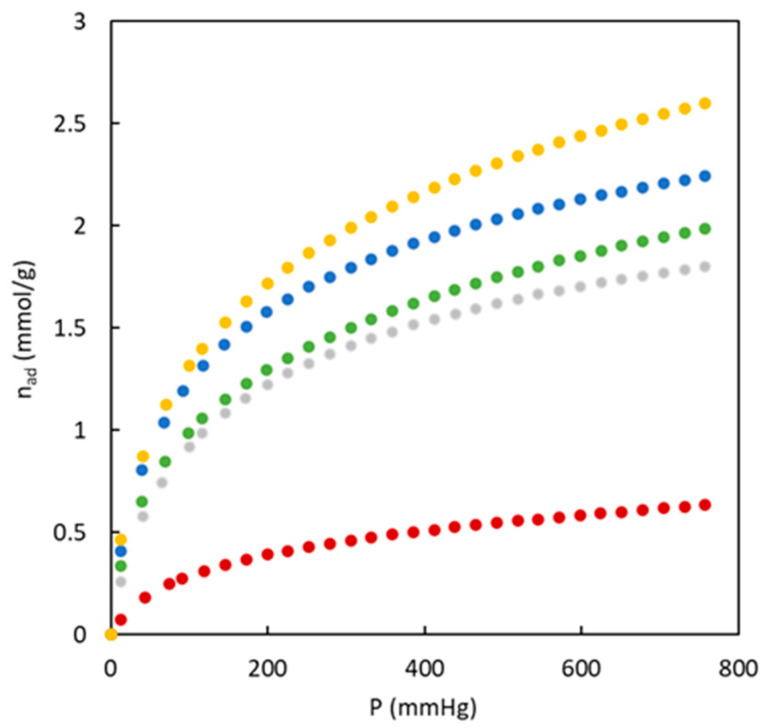
Adsorption isotherms corresponding to CO_2_ (273 K) for carbons from copolymers with and without sulfur. BC10/90-O (blue), BC26/74-O (grey), BC43/57-O (red), BC10/90-S (green), BC26/74-S (yellow).

Figure 5 shows the experimental data corresponding to CO_2_ adsorption isotherms at 273 K for the different carbons synthesized in the present work. An important difference is observed when the experimental adsorption isotherms in Figure 5 are compared with previously discussed data of nitrogen adsorption isotherms at 77 K because the differences between materials with and without sulfur are low. BC43/57-O shows the highest difference compared to the others, with a low value of adsorbed CO_2_. This behavior agrees with the microporosity percentage (Table 2) that showed the absence of this type of pores. The calculated surface area value with CO_2_ adsorption isotherm (77.6 m^2^ g^−1^) indicated that this material possesses an ultramicropore structure not detected by the N_2_ adsorption isotherm. The observed trend with the surface area due to ultramicroporosity is: BC26/74-S > BC10/90-O > BC26/74-S > BC26/74-O > BC43/57-O. This trend does not agree with the microporosity percentage as this latter is determined with N_2_ adsorption at 77 K.

**Figure 6:**
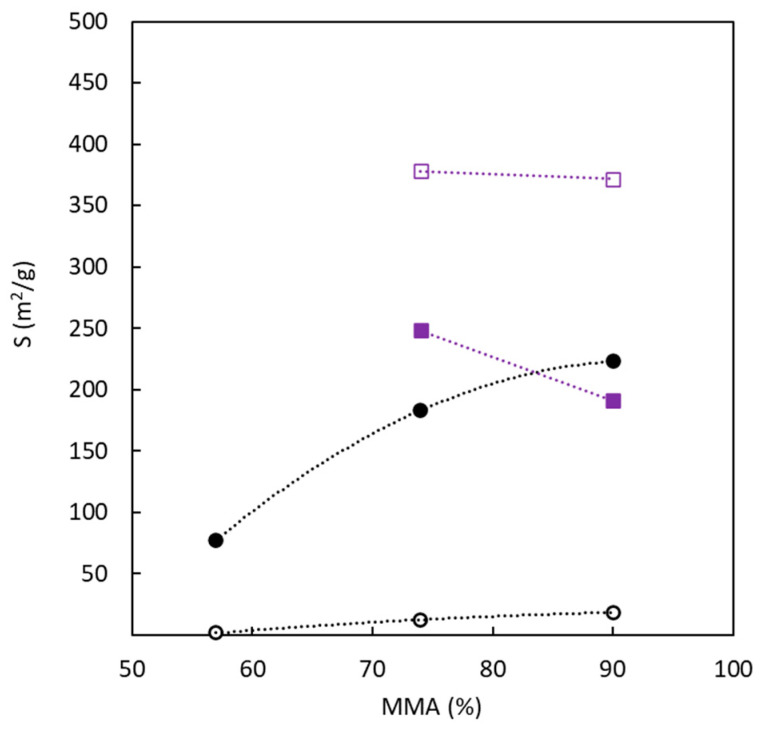
Effect of copolymer composition upon the surface area determined by the adsorption of N_2_ at 77 K and CO_2_ at 273 K. Without sulfur: (black empty circle) N_2_ at 77 K, (black full circle) CO_2_ at 273 K. With sulfur: (purple empty square) N_2_ at 77 K, (purple full square) CO_2_ at 273 K.

In general, the joint analysis of surface area determined with N_2_ at 77 K and CO_2_ at 273 K indicates that surface area due to ultramicroporosity (pore size lower than 0.7 nm) is similar for the main part of carbons with or without the use of sulfur. The main difference is observed in the surface area generated by pores below 0.7 nm. Figure 6 analyses the influence of BC composition (in terms of MMA molar amount, expressed as a percentage) in the precursor on the surface area determined for both adsorbates. The surface area determined with N_2_ at 77 K shows a very important influence of the sulfur presence and a low contribution of the copolymer composition. On the other hand, in the presence of sulfur, the MMA molar content in the precursor significantly affects the surface area values determined from CO_2_ adsorption at 273 K. In the absence of sulfur, an increase in the amount of PMMA tends to increase the ultramicroporosity. The opposite behavior is observed for sulfur-doped carbons. Concerning the surface area’s magnitude, using an MMA percentage higher than 70% allows for reaching similar values, notwithstanding the use of sulfur in the production process. Taking into account the previously discussed results, it is possible to conclude that the copolymer composition has a higher influence on the ultramicroporous structure and the surface area generated by this type of pores. Table 2 also allows us to evaluate the role of medium pore size on the surface area, which decreases with the increase in pore size.

For the influence of S-doping on the porosity > 0.7 nm, it has already been presumed by Fecher et al. [39] that the formation of S-containing groups during graphitization preferably occurs on thermodynamically more stable surface termination sites of graphitic structures (Scheme S2). Because of their bigger dimensions concerning carbon atoms, sulfur atoms tend to protrude from the graphite layers, increasing the interlayer distance and the size of the crystallites, therefore developing additional microporosity (mainly related to supramicropores 0.7 nm < D_p_ < 2 nm). A further hypothesis to support the higher values of the porosity may be given to the presence of the larger and polarizable *d* orbitals in the sulfur, where the lone pair of electrons may interact with the oxygen to strengthen the adsorption of the CO_2_ used to get the specific measurement [36]. These previous conclusions are in agreement with the experimental results (Table 2) because carbons with sulfur reach similar values of ultramicroporosity, but in addition, they reach an enhancement in supramicroporosity (determined with N_2_ at 77 K) in comparison with carbons without sulfur.

The comparison of present results with the characteristics of carbons generated from PAN homopolymers [26] allows us to conclude that the largest influence is related to the porous structure with D_p_ > 0.7 nm and mainly in S-doped material that enhances this type of porosity (supramicropores and mesopores). These conclusions agree with those concerning the role of a sacrificial component in this type of material that tries to increase mesoporosity [9].

**Figure 7:**
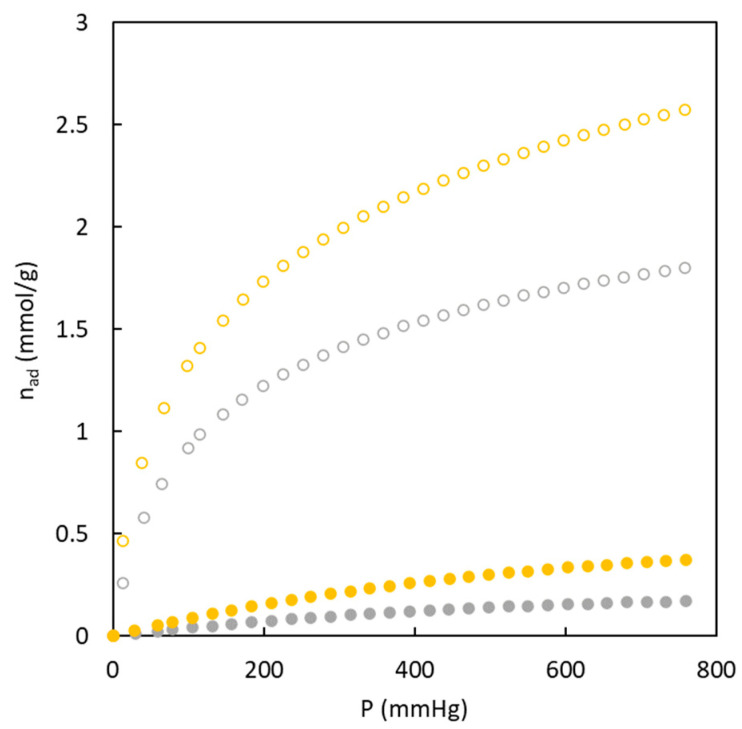
Adsorption isotherms experimental data for N_2_ (full symbols) and CO_2_ (empty symbols) at 273 K. BC26/74-O(grey), BC26/74-S (yellow).

The suitability of the carbons prepared in the present work for separating gas streams by adsorption was assessed; in particular, a gas stream with a mixture of CO_2_ and N_2_ corresponding to a post-combustion stream was considered. To carry out this type of evaluation, CO_2_ and N_2_ isotherms at 273 K were compared; an example of the obtained experimental results is shown in Figure 7. 

These experimental data show that the carbons have a higher affinity for CO_2_ molecules, reaching values ten times higher than N_2_ adsorption. This behavior is interesting to be used as an adsorbent to reach a suitable separation. Figure 7 also shows the role of sulfur in the adsorption isotherms of both gases. It can be seen that the presence of sulfur enhances the adsorption of both gases. On the one hand, the greater nitrogen adsorption may be related to the high surface area previously determined (368.6 m^2^ g^−1^). In comparison, the higher carbon dioxide adsorption may be produced by the joined effect of higher ultramicropore surface area (249.9 m^2^ g^−1^) and the affinity of this gas for sulfur, which agrees with previous studies [26].

The experimental data of adsorption isotherms for both gases employed in the present work were used to calculate the apparent selectivity value using Equation (1): this way, it was possible to compare the suitability of these carbons for nitrogen purification from a mixture containing 15 mol.% CO_2_ and 85 mol.% N_2_, which usually identifies a post-combustion stream composition.

(1) SCO2/N2=nCO2PCO2nN2PN2

where *n_CO_*_2_ and *n_N_*_2_ are the adsorbed amounts of the two gases, and *P_CO_*_2_ and *P_N_*_2_ correspond to the partial pressure of carbon dioxide and nitrogen, respectively.

**Figure 8:**
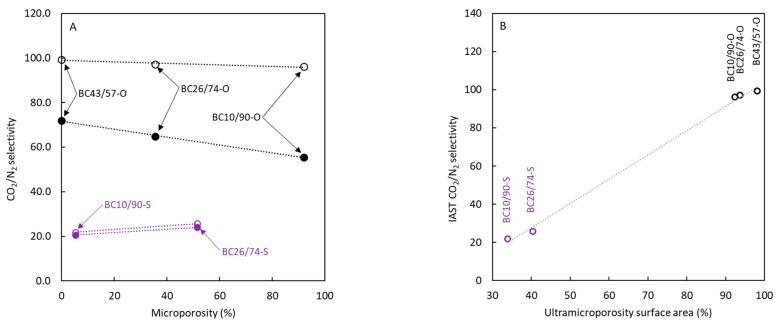
Influence of microporosity, determined with N_2_ at 77 K (**A**), and ultramicroporosity, determined by comparing surface area determined with N_2_ at 77 K and CO_2_ at 273 K on CO_2_ selectivity data (**B**). (full symbols) apparent selectivity, (empty symbols) IAST selectivity.

Figure 8A shows the calculated values of CO_2_/N_2_ apparent selectivity versus each carbon’s microporosity percentage. The analysis of the influence of this solid characteristic on CO_2_ adsorption (and selectivity in this case) can be interesting because several studies [38,40,41] have indicated its possible influence on adsorption selectivity when different gases are employed. Figure 8A shows that the influence of microporosity has a low effect, probably due to the absence of ultramicroporosity presence. The most important result observed for apparent selectivity data is the important enhancement in CO_2_ selectivity with the absence of sulfur in carbons that can be related to the negligible surface area corresponding to supramicropores and mesopores (surface area determined with N_2_ at 77 K) and the main presence of ultramicropores. Several authors considered that the estimation of CO_2_ selectivity can be improved using the ideal adsorption solution theory (IAST) [42] because this thermodynamic analysis allows taking into account a certain degree of competition between molecules for the active sites in the solid surface. This theory analyses the gas-solid adsorption equilibrium based on vapor-liquid equilibrium concepts [43,44].

The calculated CO_2_ selectivity data using IAST are also included in Figure 8 (empty symbols). The trends about the influence of microporosity on IAST selectivity that were obtained are similar to those previously discussed for apparent selectivity. Concerning the magnitude of this parameter, IAST allows us to estimate values of CO_2_ selectivity higher than those corresponding to apparent selectivity. This difference agrees with the effect of competitive adsorption between considered gases. The increase in IAST values for sulfur-doped carbons is negligible, but the difference is high for non-doped materials, approaching values close to 100. Specifically for non-doped carbons, IAST calculations indicate that a purity in adsorbed molecules close to 95% CO_2_ can be achieved. 

The calculated values using IAST indicate no differences between selectivity values for non-doped materials. This finding can be attributed to the very low surface area associated with large micropores and mesopores; further, the effect on IAST selectivity caused by the presence of ultramicroporosity is similar for all non-doped materials, though the CO_2_ adsorption could be different.

The positive influence of microporosity upon CO_2_ adsorption has been established in the last few years but Figure 8A does not agree with it. To better elucidate the influence of low-size pores upon selectivity, plot B is included in Figure 8. This figure only takes into account the surface area produced by ultramicropores (pore diameters lower than 0.7 nm), and then the results support the previous discussion, highlighting a clear enhancement of selectivity with the surface area contributed by the low-size pores. The importance of ultramicropores in selectivity is related to the higher quadrupole moment and lower kinetic diameter of carbon dioxide in comparison with nitrogen. Both parameters make ultramicropores a suitable environment for carbon dioxide adsorption, reducing the presence of nitrogen molecules in these pores.

**Figure 9:**
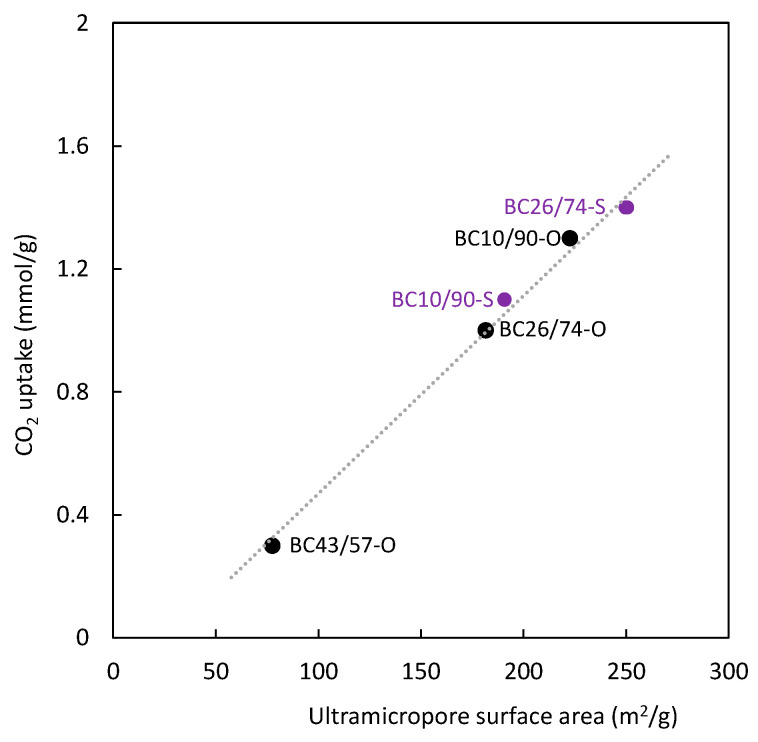
Relation between ultramicroporous surface area (determined with CO_2_ at 273 K) and CO_2_ uptake. T = 273 K. P = 15 kPa.

Considering that CO_2_ uptake and selectivity are important parameters to assess the suitability of adsorbents for industrial applications, Table 2 also includes information about the amount of CO_2_ adsorbed at 15 kPa. The experimental data corresponding to this parameter show its direct relation (see Figure 9) with the surface area corresponding to ultramicroporosity (determined with CO_2_ at 273 K). However, this relation was not observed for large micropores and mesopores (determined with N_2_ at 77 K). Therefore, it is possible to conclude that ultramicroporosity has the most important role in the CO_2_ separation process by adsorption. The CO_2_ uptake and selectivity results indicated that BC 10/90-O carbon possesses suitable characteristics for CO_2_ separation from a typical post-combustion gas stream.

The previous results’ discussion about carbon dioxide uptake and selectivity allows us to conclude the high importance of some porous characteristics of sorbents, such as surface area (mainly ultramicropore one), pore size, and pore volume. In addition, the presence of heteroatoms can provide suitable surface characteristics in order to adsorb CO_2_. Carbons fabricated in the present work have shown a slight increase in the presence of N in BC-O materials while sulfur was detected in BC-S ones. The enhancement of CO_2_ adsorption was not clearly detected by the presence of these heteroatoms [45,46], but the presence of sulfur causes an increase in the size of pores, increasing the CO_2_ uptake and reducing the selectivity. 

The higher weight of ultramicroporosity upon CO_2_ selectivity is supported by previous conclusions about the important role of Van der Waals and electromagnetic interactions in this type of pores in agreement with the physical type adsorption [47]. 

Table 4 reviews CO_2_ uptake and selectivity obtained for different carbonaceous materials from different precursors. On such basis, it may be observed that the carbons produced with an activation step tend to generate a high carbon dioxide uptake but with relatively low selectivity values. Only materials fabricated from graphene oxide and carbon nanofibers can reach interesting values for both parameters. A negative issue of these materials is related to the complex synthesis procedure in comparison with other materials.

In general, the most promising carbon, i.e., BC10/90-O, synthesized in the present work, shows interesting CO_2_ uptake and selectivity values. It is worth noting that the materials used in the present work have not been activated; for this reason, the surface area is low, and activated carbons can reach higher values of CO_2_ uptake. On the other hand, the activation procedure is an energy-intensive process that increases both the cost of the adsorbent and the carbon footprint.

**Figure 10:**
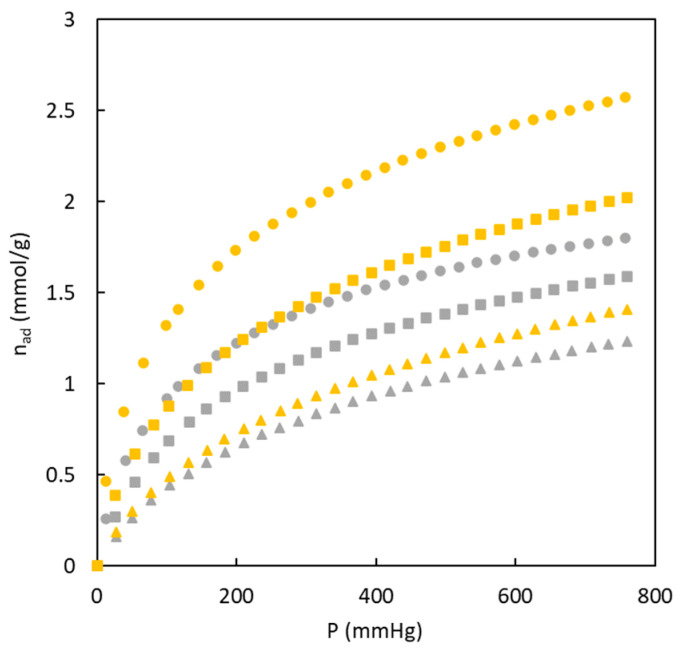
Effect of temperature upon the CO_2_ adsorption isotherms. BC26/74-S (yellow), BC26/74-O (grey). 273 K (circle), 298 K (square), 313 K (triangle).

This work also analyzes the influence of temperature on CO_2_ adsorption isotherms. Figure 10 shows some examples of the gas adsorption experimental results for this type of analysis. In all cases, a temperature increase favors the decrease in the amount of CO_2_ adsorbed, which agrees with the existence of physical adsorption. In order to use these materials for gas separation operation (specifically for CO_2_ separation) by adsorption, chemical adsorption must be avoided to reduce the cost associated with sorbent regeneration. 

The experimental data displayed in Figure 10 also highlight that the effect of temperature on the amount of CO_2_ adsorbed is different for both materials (BC 26/74-O and BC 26/74-S), and this difference could affect the energy involved in the adsorption process.

**Figure 11:**
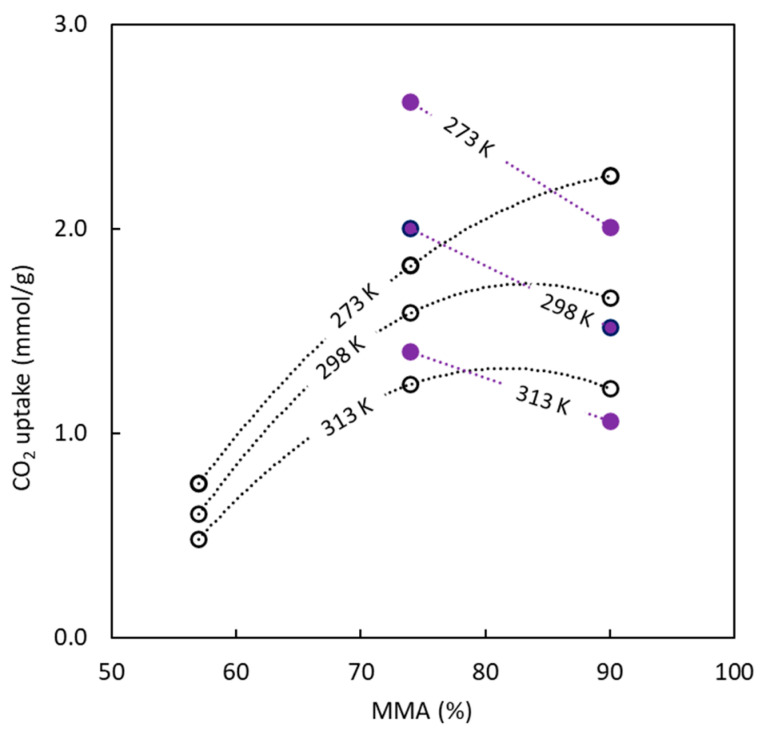
Influence of copolymer composition used in the carbon precursor and temperature upon the CO_2_ uptake at P = 101.3 kPa = 760 mmHg. Full symbols correspond to BC-S series; empty symbols correspond to BC-O series.

In relation to the influence of copolymer composition upon CO_2_ uptake, Figure 11 tries to explain the effect of temperature and copolymer composition. For all adsorbents, an increase in temperature causes a decrease in the amount of CO_2_ adsorbed, but the effect caused by BC composition shows different behaviors. The sulfur-doped carbons show a decrease in the CO_2_ uptake values with MMA concentration, unlike the materials synthesized without sulfur, which show the opposite behavior. These trends are influenced by the pore size that sacrificial PMMA generates. For materials produced in the presence of sulfur, an increase in MMA percentage in the precursor increases the pore size while lowering the CO_2_ uptakes (previously discussed). The opposite effect is observed for carbons obtained without sulfur.

The experimental data corresponding to CO_2_ adsorption isotherms were exploited to analyze the behavior of some widely used equations (Langmuir, Freundlich, and Toth) due to their simplicity and because they have shown good results. Table 5 displays the fitting parameters corresponding to each model and the sum of square errors for the different series of experimental data.

**Figure 12:**
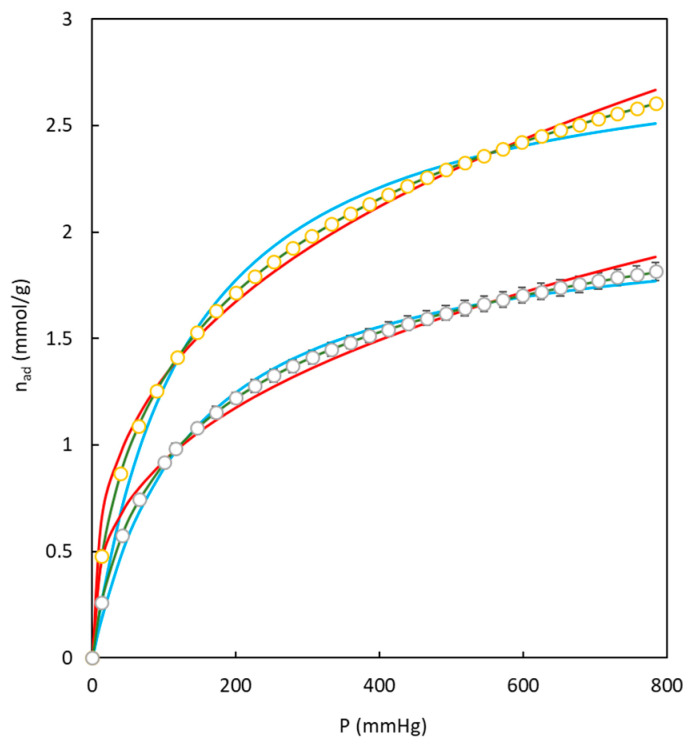
Examples of the fitting behavior of models used in present work for CO_2_ adsorption experimental data at 273 K. BC26/74-S (◯, yellow), BC26/74-O (◯, green). Langmuir (blue), Freundlich (red), Toth (green).

Figure 12 compares the behavior of each model to fit the experimental data of adsorption isotherms corresponding to two different carbons employed in the present work. Figure 12 and Table 5 demonstrate that the Toth model better fits the experimental data, while Langmuir and Freundlich models reach higher deviations. 

Toth model includes in its equation the t parameter that allows us to evaluate the homogeneity degree of a carbon surface [57]. If *t* takes values closer than 1, the Toth model is transformed on the equation developed by Langmuir. On this basis, it is possible to conclude that the material surface possesses homogeneous characteristics. The carbons synthesized in the present work showed *t* values far from 1 for CO_2_ adsorption. For this reason, an important degree of surface heterogeneity can be present in these materials. The sulfur-doped carbons showed *t* values far from 1. These results agree with the previously analyzed data that show an increase in the functional groups with the presence of sulfur atoms in the carbon structure [26]. This conclusion agrees with the fact that for sulfur-doped carbons, the Freundlich model shows a better fit than the Langmuir equation. Then, for this type of carbon, both physisorption and chemisorption might contribute to carbon dioxide adsorption.

The experimental data corresponding to CO_2_ adsorption isotherms at different temperatures were used to calculate the heat of adsorption. The Clausius-Clapeyron equation (Equation (2)) has been used, assuming that this parameter is not dependent on temperature [58].

(2) $$ln\;P=A-\frac{Q_{st}}{R}\cdot \frac{1}{T}$$

where *Q_st_* (kJ mol^−1^) is the heat of adsorption, *R* is the ideal gas constant (8.314 J mol^−1^ K^−1^), *θ* is the CO_2_ surface coverage at a pressure *P* (Pa) and temperature *T* (K).

**Figure 13:**
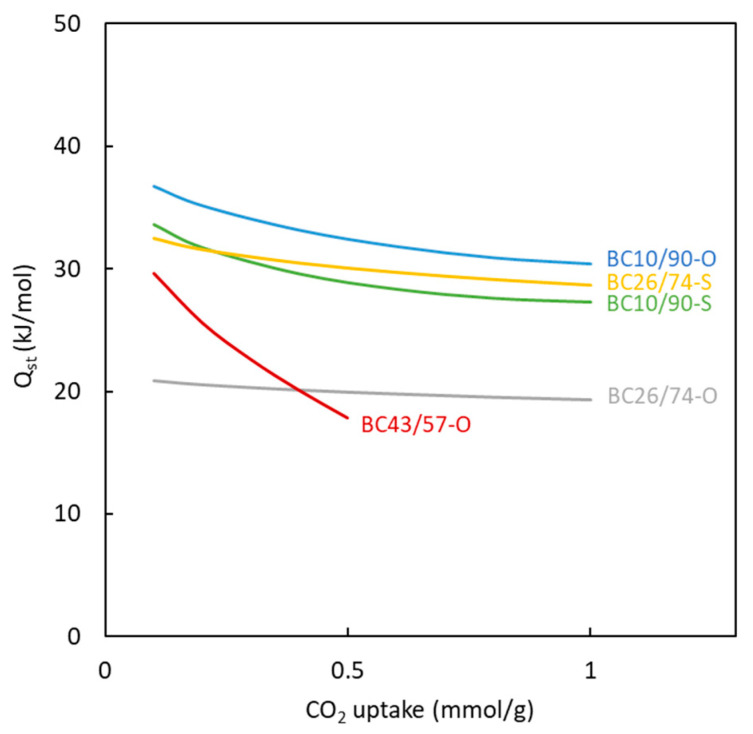
Effect of material and surface coverage on the value of heat of adsorption.

To determine the heat of adsorption at different CO_2_ uptakes, suitable modeling of the influence of pressure on the amount of CO_2_ adsorbed is needed. The Toth model was chosen based on the results shown in Table 5. The values of heat of adsorption calculated for the different materials and at different degrees of surface coverage are shown in Figure 13. These data allow us to conclude that the magnitude of the heat involved in the adsorption of CO_2_ molecules for all the carbons is 20–35 kJ mol^−1^. The magnitude of this parameter allows us to confirm that the adsorption type is physical, in agreement with the same conclusion reached when the influence of temperature on the amount of CO_2_ adsorbed was investigated.

Considering the influence of the surface coverage by the adsorbed CO_2_, a decrease in the heat of adsorption value is observed for all materials. This type of behavior has commonly been observed [59,60] for carbons. It is attributed to the energetic heterogeneity of the surface of the carbons. At low CO_2_ coverage (i.e., low amount of CO_2_ adsorbed onto the surface), the molecules are mainly adsorbed in the ultramicropores, showing the most intense interactions between adsorbate and adsorbent. This behavior again agrees with the values of the t parameter of the Toth model (far from 1).

A comparison between the calculated results corresponding to the different synthesized carbons shows similar behavior for most of them. BC10/90-O carbon previously exhibited an important microporosity degree that can be attributed to the high heat of adsorption. On the other hand, the lowest heat of adsorption value (BC26/74-O) is reached for carbon with a low degree of microporosity. BC26/74-O carbon shows a high influence of adsorbed CO_2_ on the heat of adsorption. This finding is due to the presence of only ultramicroporosity in the solid, which contributes to large interactions between adsorbate and adsorbent.

All the sulfur-doped carbons show similar adsorption behaviors. BC10/90-S carbon shows a low amount of microporosity, but the heat of adsorption is similar to most of the carbons. This behavior can be due to the presence of functional groups in all the doped carbons generated by sulfur, which increase the intensity of chemical interactions with CO_2_ and are not affected by both the surface coverage and the microporosity degree. This behavior agrees with the previous discussion about the value of the t parameter of the Toth model for sulfur-doped materials.

## 4. Conclusions

The characterization of the porous structure of the synthesized carbon materials has indicated that the presence of sulfur tends to modify the structure of the carbons from microporous to mesoporous. A more detailed analysis showed no clear influence of precursor composition on the surface area generated by ultramicropores; conversely, the presence of sulfur enhances the surface area produced by the supramicropores and mesopores.

The use of PAN-*b*-PMMA BCs with a range of molar composition between 10/90 and 47/53 as precursors allows the obtainment of carbons with different degrees of porosity; taking into account their pore size, the different materials can show suitable characteristics for their use in gas separation units, reaching adequate gas adsorption values (taking into account the absence of activation procedure) and high selectivity towards CO_2_ (enhanced by the degree of ultramicroporosity). The best selectivity and CO_2_ uptake values achieved by BC10/90-O and BC26/74-S materials agree with the values of heat of adsorption since the latter indicates a higher degree of interaction between gas molecules and the surface. In any case, the range of values of this parameter allows us to conclude that the adsorption type is physical in all the investigated cases.

These findings confirm the role of PMMA as a sacrificial block that may only affect the morphology of carbons stabilized in oxygen, inducing their nanostructuration, but not changes in their chemical composition.

## Figures and Tables

**Table 1:** Composition and molecular characteristics of PAN-*b*-PMMA precursors.

Designation	PAN M_n_ (kDa) ^a^	PMMA M_n_ (kDa) ^a^	Molar Composition (AN/MMA) ^a^	φPAN ^b^
PAN	1.3	-	100/0	1
BC10/90	1.3	23.0	10/90	0.10
BC26/74	1.3	7.2	26/74	0.26
BC43/57	1.3	3.3	43/57	0.44

^a^ by 1H NMR. ^b^ volume fraction calculated considering the following density (g cm^−3^): PAN = 1.183, PMMA = 1.159.

**Table 2:** Char yields and textural characterization by N_2_ (77 K) and CO_2_ (273 K) adsorption isotherms and CO_2_ uptake of carbons.

Carbon	Char Yield	S_N2_ ^a^	V_T_ ^b^	V_MES_ ^c^	V_MIC_ ^d^	Microporosity	D_P_ ^e^	S_CO2_ ^a^	Uptake ^f^
	(wt.%)	(m^2^ g^−1^)	(cm^3^ g^−1^)	(cm^3^ g^−1^)	(cm^3^ g^−1^)	(%)	(nm)	(m^2^ g^−1^)	(mmol g^−1^)
BC10/90-O	5.9	18.3 ± 0.3	0.0089	0.0007	0.0082	92.1	2.0	222.7 ± 2.3	1.3
BC26/74-O	16	12.3 ± 0.1	0.0091	0.0059	0.0032	35.2	3.0	181.7 ± 2.2	1.0
BC43/57-O	24	1.5 ± 0.1	0.0029	0.0029	0.0000	0.0	7.9	77.6 ± 1.1	0.3
BC10/90-S	6.2	371.6 ± 2.2	0.7411	0.7023	0.0388	5.2	8.0	190.8 ± 3.2	1.1
BC26/74-S	15	368.6 ± 4.8	0.2527	0.1227	0.1300	51.4	2.7	249.9 ± 4.4	1.4

^a^ S: surface area; ^b^ V_t_: total pore volume; ^c^ V_mes_: mesoporous volume; ^d^ V_mic_: microporous volume; ^e^ D_p_: pore diameter; ^f^ determined at 15 kPa.

**Table 3:** Average composition of carbons and main functional groups (C-, N- and S-containing species) as determined by XPS.

Carbon Series	C (wt.%; Bond Types)	N (wt.%; Bond Types)	O (wt.%)	S (wt.%; Bond Types)
BC-O	81; C=C, C-H, C-O, C=O	13; pyridinic, pyrrolic	6	-
BC-S	80; C=C, C-H, C-S	12; pyridinic, pyrrolic	5	3; C-S-C, C-S(O)_2_-C

**Table 4:** Literature data corresponding to CO_2_ uptake and selectivity for some carbonaceous adsorbents.

Adsorbent (Precursor) ^a^	Conditions ^b^ T(K)/t(min)	CO_2_ Uptake (mmol g^−1^)	CO_2_/N_2_ Selectivity	Refs.
AC (peanut shell)	C-823/30 A-953/90/KOH	7.3	7.9	[48]
AC (rice husk)	C-793/20 A-1053/60/KOH	6.2	19.9	[49]
AC (bamboo)	C-773/90 A-873/90/KOH	7.0	10.2	[50]
OMC (Pluronic F127)	C-623/300	3.0	12.8	[51]
OMC (N-dopped Pluronic F127)	C-673/120 A-1123/120/KOH	4.9	14.4	[52]
GO (N-dopped)	C-1073/120	6.5	454	[53]
CNF (PAN)	C-873/180	2.3	95	[54]
CMF (PAN)	C-1173/90	3.4	18	[55]
AC (PBZC)	C-773/60 A-873/60/KOH	6.67	35	[56]

^a^ AC: activated carbon; OMC: Ordered Mesoporous Carbon; GO: Graphene Oxide; CNF: Carbon Nanofibers; CMF: Carbon Microfibers; PBZC: Polybenzoxazine. ^b^ Conditions refer to carbon formation (C) and activation (A).

**Table 5:** Fitting parameters corresponding to CO_2_ adsorption for Langmuir, Freundlich, and Toth equations.

		Langmuir			Freundlich			Toth			
Carbon	T (K)	n_m,L_	K_L_	SSE_L_	n_m,F_	K_F_	SSE_F_	n_m,T_	K_T_	t	SSE_L_
BC10/90-O	273	2.485	0.0095	0.081	3.211	0.2926	0.123	3.586	0.0228	0.48	0.0002
BC10/90-O	298	2.078	0.0047	0.022	2.328	0.0992	0.041	3.306	0.0063	0.53	0.0003
BC10/90-O	323	1.899	0.0023	0.003	1.762	0.0291	0.018	2.791	0.0021	0.67	0.0002
BC26/74-O	273	2.067	0.0761	0.033	2.903	0.1895	0.093	2.810	0.0132	0.55	0.0002
BC26/74-O	298	1.971	0.0049	0.017	2.387	0.1018	0.044	2.891	0.0065	0.57	0.0003
BC26/74-O	323	1.737	0.0030	0.006	1.963	0.0433	0.020	2.754	0.0031	0.59	0.0001
BC43/57-O	273	0.852	0.0074	0.011	2.882	0.0765	0.009	1.505	0.0193	0.41	0.0001
BC43/57-O	298	0.779	0.0059	0.014	2.579	0.0514	0.007	2.110	0.0175	0.32	0.0019
BC43/57-O	323	0.756	0.0025	0.004	1.822	0.0136	0.002	4.742	0.0018	0.29	0.0007
BC10/90-S	273	2.276	0.0071	0.099	2.839	0.1958	0.042	5.262	0.0262	0.33	0.0004
BC10/90-S	298	1.927	0.0073	0.029	2.272	0.0838	0.018	4.622	0.0066	0.39	0.0001
BC10/90-S	323	1.569	0.0026	0.006	1.847	0.0299	0.010	3.187	0.0023	0.51	4 × 10^−5^
BC26/74-S	273	2.922	0.0077	0.170	2.935	0.2754	0.080	6.194	0.0289	0.34	8 × 10^−6^
BC26/74-S	298	2.95	0.0075	0.147	2.878	0.266	0.077	6.161	0.0252	0.35	7 × 10^−6^
BC26/74-S	323	2.005	0.0027	0.014	1.891	0.0423	0.012	5.253	0.0025	0.43	4 × 10^−5^

n_m,L_ and n_m,T_ (mmol g^−1^); K_L_ (mmHg^−1^); K_F_ (mmol mmHgnF g^−1^); K_T_ (mmHg^−1^); n_F_, t and SSE are dimensionless parameters.

## Data Availability

Data is available upon request from the corresponding author.

## Supplementary Materials

The following supporting information can be downloaded at: https://www.mdpi.com/article/10.3390/polym16020269/s1, Scheme S1. Schematic illustration of the formation of graphitic carbon from a PAN-based BC precursor; Scheme S2. Possible positions of sulfur incorporated into the graphitic carbon network; Figure S1. Raman spectra of BC10/90-O (solid line) and BC10/90-S (dashed line) in the 900–1900 cm^−1^ range. Figure S2. Pore size distribution of BC10/90-O and BC10/90-S.
