# Supplementary material for: S/N/O-Enriched Carbons from Polyacrylonitrile-Based Block Copolymers for Selective Separation of Gas Streams
Source: Polymers (Basel). 2024 Jan 18;16(2):269. doi: 10.3390/polym16020269 (PMC10819996; doi:10.3390/polym16020269)
Supplement: Supplementary file 1 [file polymers-16-00269-s001.zip › polymers-2816181-supplementary.pdf]

S/N/O-Enriched Carbons from Polyacrylonitrile-Based Block Copolymers for Selective Separation of Gas Streams

Diego Gómez-Díaz [1,*], Lidia Domínguez-Ramos [1,2,3], Giulio Malucelli [4], María Sonia Freire [1], Julia González-Álvarez [1] and Massimo Lazzari [2,3,*]

[1] Departamento de Ingeniería Química, ETSE, Universidade de Santiago de Compostela, Rua Lope Gómez de Marzoa s/n, 15782 Santiago de Compostela, Spain; lidia.dominguez2@usc.es (L.D.-R.); mariasonia.freire@usc.es (M.S.F.); julia.gonzalez@usc.es (J.G.-Á.)
[2] Departamento de Química Física, Facultade de Química, Universidade de Santiago de Compostela, Avenida das Ciencias s/n, 15782 Santiago de Compostela, Spain
[3] Centro Singular de Investigación en Química Biolóxica e Materiais Moleculares (CiQUS), Universidade de Santiago de Compostela, 15782 Santiago de Compostela, Spain
[4] Department of Applied Science and Technology, Politecnico di Torino, Viale Teresa Michel 5, 15121 Alessandria, Italy; giulio.malucelli@polito.it
* Correspondence: diego.gomez@usc.es (D.G.-D.); massimo.lazzari@usc.es (M.L.)

Supplementary Materials: Scheme S1. Schematic illustration of the formation of graphitic carbon from a PAN-based BC precursor; Scheme S2. Possible positions of sulfur incorporated into the graphitic carbon network; Figure S1. Raman spectra of BC10/90-O (solid line) and BC10/90-S (dashed line) in the 900–1900 cm^{-1} range. Figure S2. Pore size distribution of BC10/90-O and BC10/90-S.

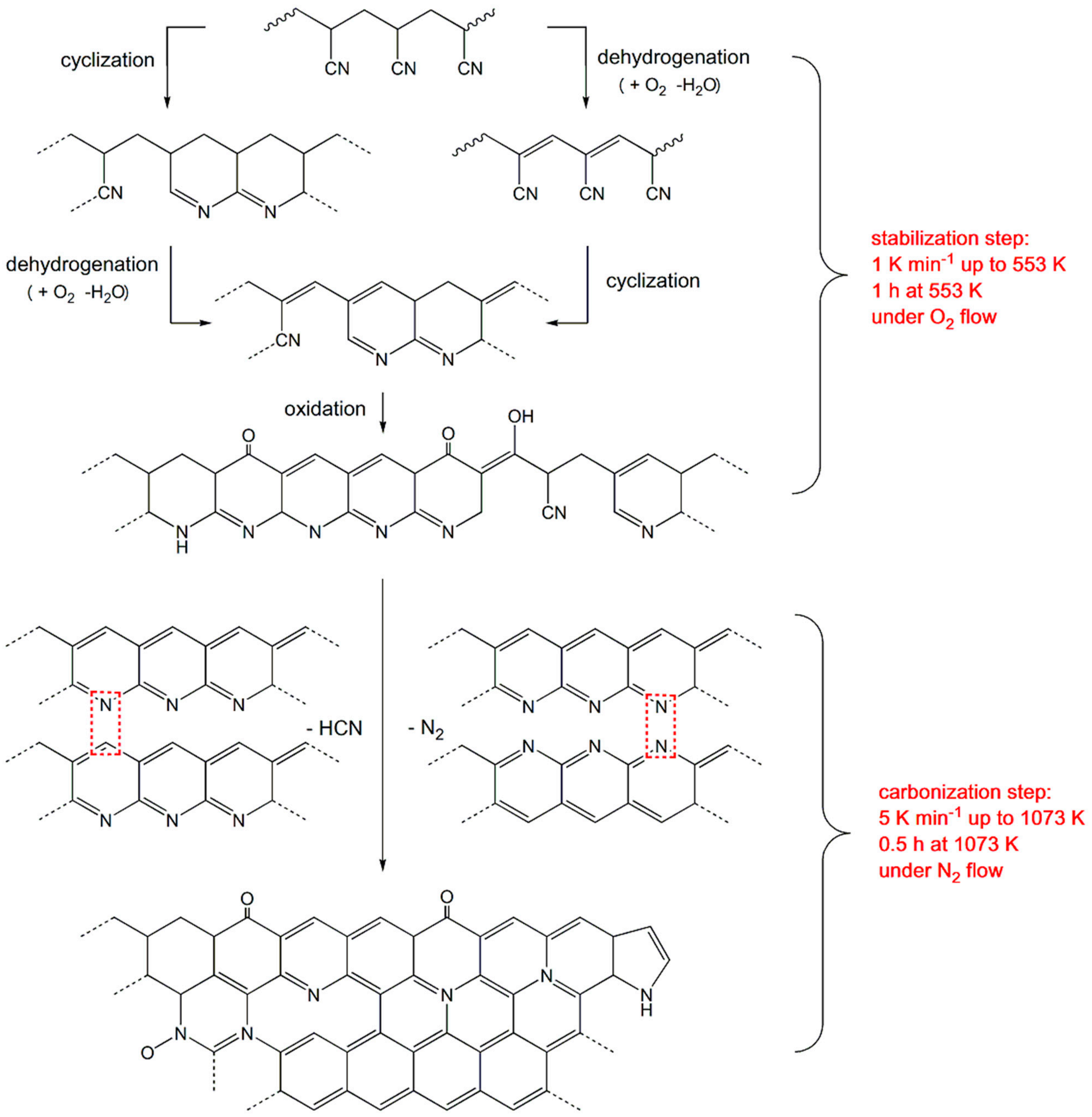

Scheme S1. Schematic illustration of the formation of graphitic carbon from a PAN-based BC precursor, also including the information resulting from XPS measurements (see examples in Figure 2 and corresponding discussion). PMMA decomposition is not reported, as it essentially consists of volatilization by unzipping. Adapted from: Fitzer, E. *Carbon* **1989**, *27*, 621–645.

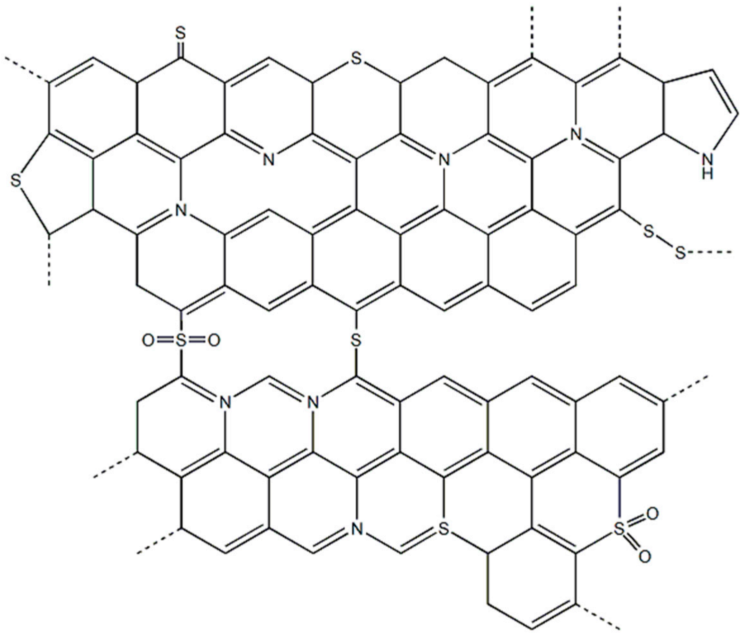

Scheme S2. Tentative positions of sulphur incorporated into the graphitic carbon network, taking into account the information resulting from XPS measurements (see examples in Figure 2 and corresponding discussion). Adapted from: Kiciński, W.; Szala, M.; Bystrzejewski, M. *Carbon N. Y.* **2014**, *68*, 1–32.

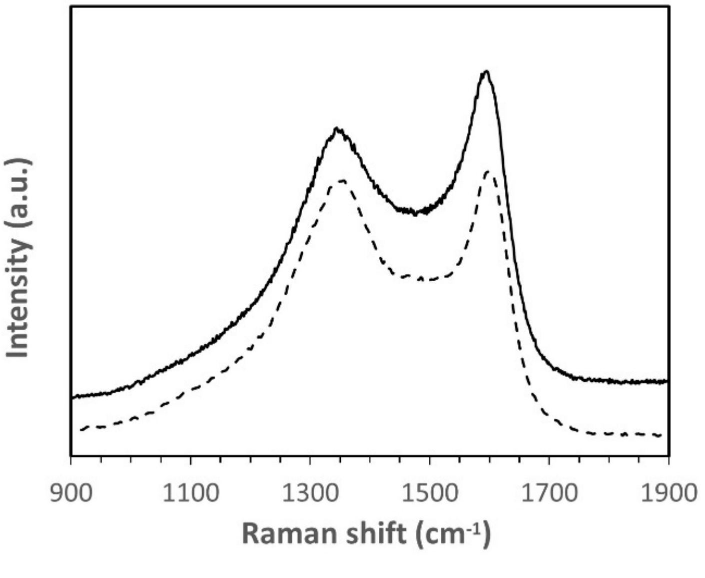

Figure S1. Raman spectra of BC10/90-O (solid line) and BC10/90-S (dashed line) in the 900-1900 cm^{-1} range.

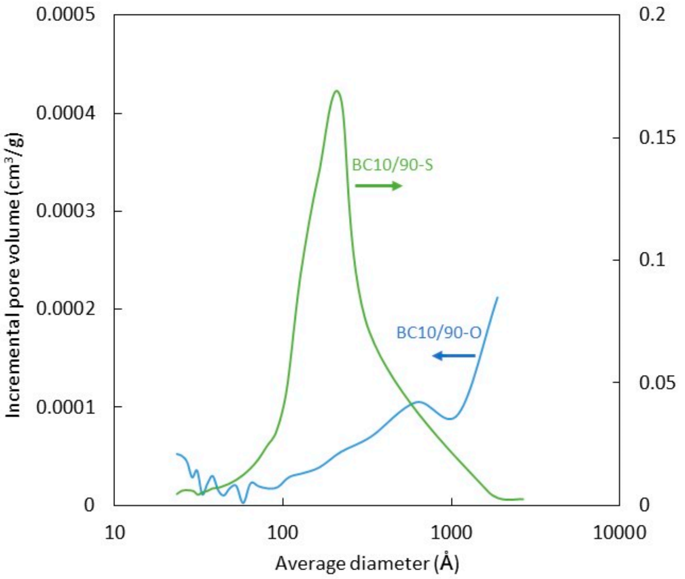

Figure S2. Pore size distribution of BC10/90-O and BC10/90-S.
